# Time-course of the DSM-5 cannabis withdrawal symptoms in poly-substance abusers

**DOI:** 10.1186/1471-244X-13-258

**Published:** 2013-10-12

**Authors:** Morten Hesse, Birgitte Thylstrup

**Affiliations:** 1 Centre for Alcohol and Drug Research, Aarhus University, Artillerivej 90, 2nd, 2300 Copenhagen S, Denmark

**Keywords:** Cannabis withdrawal, Withdrawal symptoms, Poly-substance abusers

## Abstract

**Background:**

Evidence is accumulating that a cannabis withdrawal syndrome is common, of clinical significance, and has a clear time course. Up till now, very limited data exist on the cannabis withdrawal symptoms in patients with co-morbid substance use disorders, other than cannabis use and tobacco use.

**Methods:**

Symptoms of withdrawal were assessed through patient self-reports during detoxification in Danish residential rehabilitation centers. Patients (n = 90) completed booklets three times during their first month at the treatment centre. Self-reported withdrawal symptoms was rated using the DSM-5 Withdrawal Symptom Check List with withdrawal symptoms from all classes of substances, with no indication that the described symptoms should be attributed to withdrawal. Self-reported time since last use of cannabis was used as a predictor of cannabis withdrawal severity.

**Results:**

With the exception of loss of appetite, time since last use of cannabis was associated with all types of withdrawal symptoms listed in the DSM-5. Only four of 19 symptoms intended to measure withdrawal from other substances were related to time since last use of cannabis, including vivid, unpleasant dreams.

**Conclusions:**

The findings yield strong support to the notion of a cannabis withdrawal syndrome, and gives further evidence for the inclusion of the criterion of vivid, unpleasant dreams. Further, the findings speak against the significance of demand characteristics in determining the course of the symptoms of cannabis withdrawal.

## Background

The cannabis withdrawal phenomenon has received growing interest in recent years. Cannabis withdrawal does not typically cause significant medical or psychiatric problems as do opioid, alcohol, or benzodiazepine withdrawal [1], but implications of withdrawal symptoms include the risk of relapse and the well-being of patients [2].

A withdrawal syndrome is characterized by onset after cessation of drug use followed by a gradual return to baseline levels, and reversed by new drug use. Until recently, no official definition of a cannabis withdrawal was listed in the official diagnostic nomenclatures ICD-10 and DSM-IV.

With the recent publication of the DSM-5, a cannabis withdrawal syndrome is now officially recognized with defined criteria [3]. In the following, we shall review the evidence for these criteria as well as comment on criteria that are listed as ancillary in the DSM-5.

### Status of the cannabis withdrawal syndrome

In 1994, Carroll and colleagues found that withdrawal was rarely endorsed by cannabis users, and Guttman scaling showed that only the most severely affected cannabis dependent patients reported cannabis withdrawal [4]. However, in the absence of specific criteria, respondents may not have interpreted symptoms that occurred after cannabis cessation as withdrawal symptoms. Later research has indeed produced a different picture [2].

Studies have found that the cannabis withdrawal syndrome appears to be comparable to the tobacco withdrawal syndrome [5,6], and is associated with severity of cannabis problems at follow-up [7,8]. Most symptoms of cannabis withdrawal increase immediately after cessation and decline over the next weeks [9-11], see however [12]. Additionally, studies have shown that the symptoms of cannabis withdrawal can be treated effectively with oral THC [13-15] and that sleep problems associated with cannabis withdrawal can be treated with zolpidem [16].

### Specific symptoms of cannabis withdrawal listed in the proposal for DSM-5

Cannabis withdrawal has been recommended for inclusion in the DSM-5 with the following symptoms: A. Cessation of heavy and prolonged cannabis use; B. 3 or more of the following developments within several days after cessation: 1. Irritability; 2. Nervousness; 3. Sleep difficulty; 4. Decreased appetite; 5. Restlessness; 6. Depressed mood; 7. Physical symptoms and discomfort.

In the following, studies that have supported the inclusion of these symptoms are presented.

*Irritability.* Most studies give strong support to the relevance of irritability and anger as a common and significant symptom of cannabis withdrawal e.g. [7,9,11,17-23]. Irritability and anger may have a later onset and a longer duration than most other withdrawal symptoms [11]. Additionally, more severe expressions of aggression, such as attacking someone physically, may occur later than milder symptoms of irritability [20].

*Nervousness*. Nervousness is also a commonly reported symptom [7,8,11,17,19-23]. Prospective research has found that nervousness associated with cannabis withdrawal is a source of significant distress [9], and that it gradually declines in severity after cannabis cessation [10,11]. However, not all studies have supported the significance of nervousness as a symptom of cannabis withdrawal e.g. [18].

*Sleep difficulty*. Sleep difficulty or insomnia is also frequently reported as a withdrawal symptoms [7-9,11,17,18,20,21], that decrease with time since cessation [10,11,18]. However, studies have found some uncertainty regarding the time course: prospective studies using self-report measures suggest that sleep problems decline fairly linearly after cessation of cannabis use [10,11], whereas a study using polysomnogram data indicated that sleep quality declined gradually over 13 days with no reversal [24].

*Decreased appetite or weight loss.* Decreased appetite or weight loss has been robustly reported across studies [9,11,18-23] with a quick onset after cessation, followed by a relatively quick reversal [11].

*Restlessness*. Restlessness has generally been supported as a common and significant symptom of cannabis withdrawal [7-11,17,20,21] with a slightly later onset than most symptoms [11].

*Depressed mood*. Depressed mood is also commonly reported, although not as often as nervousness or irritability [7,8,17,19-21,23]. Some prospective studies have failed to show that it follows a clear withdrawal pattern [11,18], but other research supports its inclusion as a valid withdrawal symptom [9].

*Physical symptoms*. Physical symptoms such as stomach pain, shakiness/tremors, sweating, fever, chills or headache have been reported at variable rates, and generally at lower rates compared with other symptoms [7,8], e.g. [17,20-23], but some research has supported most of these symptoms as indications of withdrawal [9], see however [18].

### Other symptoms

The withdrawal symptoms listed in the DSM-5 are not the only symptoms that have been studied in clinical and experimental research. Some of these other symptoms are likely to be highly correlated with symptoms in the DSM-5, and have been listed as symptoms that may also occur [3].

A frequently reported symptom is increased appetite which is about as common as decreased appetite [19-21], although Allsop and colleagues have reported that it is not valid as a withdrawal criterion [9].

One of the most commonly reported withdrawal symptoms not included in the DSM-5 is vivid unpleasant dreams, although it appears to be as common as the symptoms listed for the DSM-5 [8,9,11,17,18], see however [21]. The time course of these dreams differs from other symptoms studied with a later onset and a longer duration [11,20]. Vivid, unpleasant dreams is not easily subsumed under any of the included symptoms in the DSM-5, but it is worth noting that strange and unpleasant dreams is listed in the DSM-5 criteria for amphetamine and cocaine withdrawal.

### Methodological limitations in the existing literature

The literature described above shows a fairly consistent picture of the cannabis withdrawal symptoms that is largely in line with the proposal to include cannabis withdrawal in the DSM-5. However, the literature on cannabis withdrawal presents some methodological challenges. One potential limitation is the problem of demand characteristics. In psychology, demand characteristics refer to “the totality of cues which convey an experimental hypothesis to the subject” [25]. In this context, giving patients cues that a given instrument is designed to measure withdrawal from cannabis may prompt them to respond in ways that they perceive matches what they believe about withdrawal in general.

For instance, if patients believe that withdrawal is a temporary cluster of symptoms that begins after cessation of drug use, they may be more likely to report symptoms after cessation of use, and stop reporting these symptoms after a while. Such cues may range from having cannabis withdrawal in the heading of the questionnaire, over to asking on the same form how many days it has been since the subject last used cannabis, to having a cannabis craving scale on the same instrument. While previous literature has addressed some aspects of demand characteristics, e.g. by using filler items [9], using alternative headings, such as “Behavior Checklist” [18], or asking participants to fill in other measures simultaneously with marijuana craving questionnaires [11], all studies have been open about the study’s focus on marijuana cessation during the recruitment phase, and it has thus been clear to respondents that questions they have answered may specifically have been associated with marijuana. This includes the fact that participants have been asked to choose a quit date for marijuana, and that they have been asked if they used marijuana prior to entering the study.

No study has so far used the seven symptoms listed in the forthcoming DSM-5 together as the indicator of withdrawal. Studies have either used some of these symptoms or included other symptoms such as craving.

Finally, the cannabis withdrawal syndrome has not yet been studied in poly-substance using patients with the exception of one small cross-sectional study by Vorspan and colleagues that indicated that sleep disturbances, but not other cannabis withdrawal symptoms, were exacerbated in patients with co-morbid opioid dependence [26]. Arguably, there are good reasons for the omission of poly-substance using patient samples when establishing the validity and time course of the cannabis withdrawal syndrome. Other drugs may influence mood, physical discomfort, sleep, appetite and overall wellbeing which may well mask or exacerbate symptoms associated with cannabis withdrawal. However, poly-substance dependent patients make up a large proportion of patients in substance abuse treatment. If poly-substance abusing patients experience clinically significant cannabis withdrawal symptoms, psycho-education, behavioral and pharmacological interventions that target the cannabis withdrawal syndrome should be made available to such patients.

### Aims of the present study

The present study was part of a larger study of residential treatment in Denmark, funded by the Ministry of Social Welfare.

The aim of this study was to assess the recommended withdrawal symptoms of cannabis in the DSM-5 through patient self-reports during detoxification in Danish residential rehabilitation centers measured over 4 weeks of early treatment.

The goals were:

1. To assess the time course of the DSM-5 cannabis withdrawal syndrome in relation to time elapsed since last use of cannabis. We hypothesized that the DSM-5 symptoms would follow a curvilinear time course from cessation of cannabis use characterized by an increase in severity immediately after cessation followed by a decrease. This was conducted by administering a scale consisting of all the criteria listed in the DSM-5 for cannabis withdrawal three times over four weeks of treatment.

2. To assess the time course of each individual symptom of withdrawal, including symptoms currently listed for other substances than cannabis, controlling for use of other drugs.

Patients with poly-substance abuse were ideal for this purpose. If only cannabis users were included in the study, this would potentially be a cue that the focus of the study was cannabis-related.

## Methods

### Procedures

We used the national residential registration base in Denmark (DanRIS) to identify residential illicit drug abuse treatment institutions in Denmark. The treatment services were then contacted through the management and introduced to the details of the study, either through prepared information material, or by a visit from the researchers or other staff affiliated with the project. In total 18 treatment services agreed to participate, and a staff member was selected as contact person for the researchers and responsible for collection of data at each site. The contact person at each site received the three “booklets” that the patients were to fill out during the first, second and fourth week after beginning treatment. The three booklets all had questions on substance use, medical treatment and experience of withdrawal symptoms. To reduce demand characteristics, patients were not instructed to complete the questionnaire on the day that they last used cannabis.

Following this, staff members at each site introduced newly enrolled patients to the study. The introduction in the first booklet was used to describe that filling out the study booklets would help understanding what problems substance users had beside their substance use. There were no exclusion criteria for participating in the study. Patients were not required to be cannabis dependent for inclusion in the study. However, patients that were deemed too chaotic to fill out the booklets, even when aided by the staff members, or who did not speak Danish were not approached.

If the patients agreed to participate, they were asked to give written consent to participation in the study. Danish institutional review boards do not assess ethics in clinical studies except those involving invasive medical procedures or experimental manipulation of drug treatment. This study did not involve invasive procedures or manipulation, but consisted of filling out questionnaires, and the written consent stated that refusal to participate would not influence the treatment that the patients were receiving at present. Thus, we can think of no way in which the present study can be greatly harmful to the participants or in any way violate the Declaration of Helsinki.

### Instruments

#### ***Addiction Severity Index (ASI)***

All three booklets contained the drug and alcohol section from the Addiction Severity Index [27], and patients answered how many days had elapsed since the last time they used each substance listed. We estimated the test-retest reliability of this question by using patients who completed the questionnaire at least two times, and included only patients who had used cannabis in the past 30 days prior to their completion of the week one booklet. At the second assessment we subtracted the time elapsed between the two booklets from the time since last cannabis use for each individual. If the answer was perfectly reliable, the values would become identical (e.g., if a person completed the first booklet two days after last cannabis use and the second booklet 7 days later, the expected value would be 9). The test retest correlation of time since last cannabis was found to be acceptable (coefficient of concordance = 0.67, 95% CI = 0.51 to 0.82, Pearson r = 0.69, p < 0.001, n = 48).

#### ***Pharmacological treatment***

Since there was no standard instrument for collecting data on type of medical treatment, a questionnaire was designed for this study to collect data on what types of medical treatment the patient received at the treatment service, including average dose of each type of medicine and number of days since last medicine intake. This questionnaire was also included in all three booklets.

#### ***The DSM-5 withdrawal symptom check list***

All items were worded to reflect the withdrawal items in the DSM-5, based on the then-available criteria on the DSM-5 webpage. The symptom checklist on withdrawal symptoms contained 26 items. The instruction stated: “Right now I experience….”, followed by a list of symptoms worded after the DSM-5 criteria for withdrawal symptoms from cannabis, cocaine, amphetamine, opioids, tranquilizers and alcohol. Items such as hallucinations or seizures that should not occur while patients were in treatment were not included, since moderate withdrawal symptoms from alcohol or benzodiazepines always lead to pharmacological treatment in Denmark. Each of the 26 items was rated on a four point Likert scale from “Not at all” to “Severely”. No mention was made in the questionnaire that the items were intended to measure withdrawal, or that patients should consider the time elapsed since they quit using drugs or taking medication. The items are shown in Table 1, along with item-total correlations for the cannabis withdrawal items at week one and the percent ever endorsing the symptom. Table 1 includes only individuals who used cannabis in the past 30 days before treatment and completed more than one booklet.

**Table 1:** Items of the withdrawal scale

	**Ever rated > “Not at all”**	**Item-rest correlation**
1. I am more irritable, angry or aggressive than usually	85%	0.47
2. I am more nervous or anxious than usually	84%	0.50
3. I have trouble falling asleep	90%	0.26
4. I have no appetite or I have begun losing weight	54%	0.21
5. I am restless	97%	0.51
6. I feel depressed	84%	0.53
7. I have abdominal pain, I am sweating, have the chills, as if I had a fever, or I have a headache	81%	0.44
8. I feel tired	93%	
9. I have vivid unpleasant dreams	82%	
10. I sleep a lot	81%	
11. My appetite has increased a lot	88%	
12. I have become so restless that people notice	74%	
13. Others notice that I have become slow in my movements and when I speak	43%	
14. I yawn a lot all of the time	88%	
15. My heart is racing	56%	
16. I have a running nose	66%	
17. I am sneezing	74%	
18. I feel a pricking sensation on my skin	51%	
19. I have gooseflesh, even when it isn’t cold	65%	
20. I feel ill	63%	
21. I have spasms	31%	
22. I have sore joints or muscles	74%	
23. I have the shakes	43%	
24. I have runny eyes	49%	
25. I am nauseous	42%	
26. I have diarrhoea	31%	

Notes: items rated on a Likert scale from 0 (not at all) to 3 (severely). Item 1–7 are intended to measure DSM-5 cannabis withdrawal symptoms. Items 8–26 are intended to measure other types of symptoms, and were included in this study as distracters.

The reliabilities for the cannabis items using only respondents who reported using cannabis was 0.71 at week 1, 0.77 at week 2, and 0.77 at week 4. A factor analysis of all respondents who completed the checklist at week 1 showed that a one factor solution was superior to a two factor solution using parallel analysis as well as the Hull Method based on root mean square error of approximation. The results remained the same, whether polychoric or Pearson correlation coefficients were used to assess the factor structure.

### Analyses

To assess the time course of symptoms, fixed effects regression was used to test the effects of time elapsed since last cannabis use, controlling for time since last use of stimulants (cocaine or amphetamine), opioids (methadone, heroin or other opioids), and tranquilizers (alcohol or benzodiazepines). For cannabis, we analyzed both linear and quadratic effects of time since last use. The substances were collapsed into classes according to the types of withdrawal symptoms described in the DSM-5 [28]. The fixed effects model controls for all stable characteristics of individuals, thus, effects of covariates should be interpreted as within-subject effects.

Missing data for cannabis withdrawal items were substituted by the respondents’ mean score on all completed cannabis withdrawal items during the same assessment when calculating the full cannabis withdrawal scale. When assessing the time course of individual symptoms, observations with missing data were omitted from the analysis. Observations with missing data on time since last use of cannabis or any other substance were omitted from analyses.

To illustrate the time course and identify peak time for each symptom, locally weighted regression analysis was carried out for the full scale and for each criterion on time elapsed since last use of cannabis.

## Results

### Participants

A total of 90 patients were included from 18 residential rehabilitation centers. Participants were 20% women and 80% men, and the mean age was 29.0 (standard deviation (SD) =7.4). We compared the sample to the general population of patients in residential treatment in the DanRIS database [29], which contains the 30-day questions from the ASI. The results are shown in Table 2. We found one statistically significant difference: patients in the study sample scored higher on concerns about employment.

**Table 2:** Comparison of the study sample and other treatment admissions during the same period

	**Sample**		**Other admissions during 2010-2011**		**Statistic**
N	90		2313		Pearson χ^2^
Female gender	21%		26%		1.01
	Mean	SD	Mean	SD	Cohen’s d
Age	29.0	7.4	31.0	8.7	-0.23
ASI composite scores	N = 62^1^		N = 2096		
Drug	0.43	0.20	0.42	0.19	0.05
Alcohol	0.30	0.34	0.23	0.31	0.23
Legal	0.25	0.24	0.22	0.25	0.12
Employment	0.20	0.29	0.12	0.25	*0.32
Financial support	0.86	0.32	0.87	0.29	-0.03
Family	0.36	0.31	0.30	0.30	0.20
Social	0.24	0.27	0.23	0.27	0.04
Psychiatric	0.43	0.28	0.47	0.26	-0.15
Medical	0.36	0.37	0.36	0.38	0.00

Notes: * p < 0.05.

^1^Of the 90 patients, 28 had not completed the Addiction Severity Index during their treatment.

Cannabis was the most commonly used drug in the past 30 days, reported by 74%, followed by stimulants (45%), opioids (42%), benzodiazepines (40%), and drinking 5 or more units of alcohol per day on any day (35%). The mean number of different substances reported was 4.2 (SD = 3.2). Eleven people used only cannabis in the past 30 days, and no other illicit drugs, and did not drink more than 5 drinks containing alcohol on any day. Patients who did not use cannabis in the past 30 days were excluded from the analyses. Additionally, patients who did not complete more than one booklet were excluded from the analyses.

In all 67 patients completed on average 2.6 booklets and had on average smoked cannabis 14.4 days prior when completing the first booklet (median = 9 days, SD = 11.3). Six patients (7%) had smoked cannabis the day before completing the first booklet.

Of the patients who could be included in the study, 34% reported receiving antipsychotics during treatment, 29% reported receiving antidepressants during treatment, and 14 (21%) reported receiving both.

#### ***The DSM-5 cannabis withdrawal symptoms***

**Figure 1:**
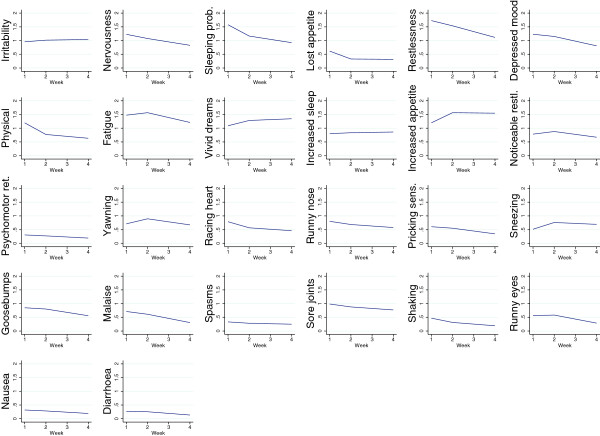
Symptom severity for each withdrawal symptom at first assessment, week 2 assessment and week 4 assessment.

The items used to assess each symptom are summarized in Table 2 along with the frequency of respondents ever endorsing the item. Means over the course of the study are shown in Figure 1. As can be seen from the graphs in Figure 1, there were some differences from week one to week two and four. Most symptoms declined slightly over time in treatment, except irritability, vivid, unpleasant dreams, increased appetite and increased sleep, sneezing, fatigue and yawning.

Typical serious opiate withdrawal symptoms such as spasms, diarrhea, nausea and shaking were rated at low severity. This may be explained by the fact that many of the opiate dependent patients were receiving either methadone or buprenorphine for tapering at the different assessment waves.

Missing items made up less than 1% of responses for any item, and missing data were substituted by means for the full-scale cannabis withdrawal scale.

**Figure 2:**
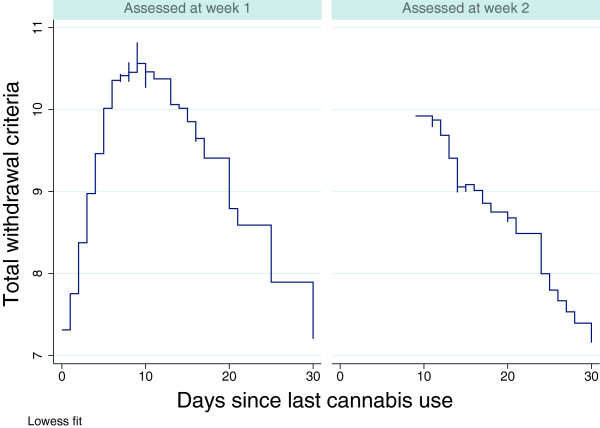
**Symptom severity for overall cannabis withdrawal syndrome as a function of time since last cannabis use.** Left-hand panel: when assessed at week 1. Right-hand panel: when assessed at week 2.

The results of the fixed effects regression for the overall DSM-5 cannabis withdrawal scale are shown in Table 3. In model 1 (linear model), cannabis symptoms decreased by 0.13 points per day elapsed since last use of cannabis. Time since last use of stimulants, opioids, alcohol or benzodiazepines was not significantly associated with cannabis withdrawal symptoms. The course of symptoms is illustrated in Figure 2. Figure 2 is divided into two, so that assessments conducted in week one are in the left panel, and assessments conducted in week two are in the right hand panel. Within each panel, severity of self-reported symptoms is shown as a function of days since last use of cannabis with locally weighted regression estimation.

**Table 3:** Fixed effects regression of the Cannabis withdrawal symptoms

	**Model 1**			**Model 2**		
	**Coef.**	**SE**	**P-value**	**Coef.**	**SE**	**P-value**
Days since last cannabis use	-0.130	0.035	0.000	0.051	0.053	0.339
Days since last alcohol or benzodiazepine use	-0.070	0.044	0.117	-0.031	0.048	0.523
Days since last opioid use	0.051	0.054	0.346	-0.061	0.044	0.175
Days since last CS use	-0.037	0.048	0.441	0.076	0.135	0.576
Days since last cannabis use squared	Not included			-0.006	0.004	0.118
Constant	11.731	1.174	0.000	10.147	1.527	0.000
Rho	0.641		0.000	0.589		0.000

Notes: CS: Central stimulants. Model 1: linear model. Model 2: quadratic model.

As can be seen, the symptom severity increased from day 1 to day 10, and dropped gradually after this. For assessments from week two, no patients reported using cannabis less than seven days ago, so only an almost linear decline is seen, following roughly the same values as in the left-hand panel. Thus, within this group, withdrawal symptoms follow a time course that is determined by days since last cannabis use, rather than time of assessment.

**Figure 3:**
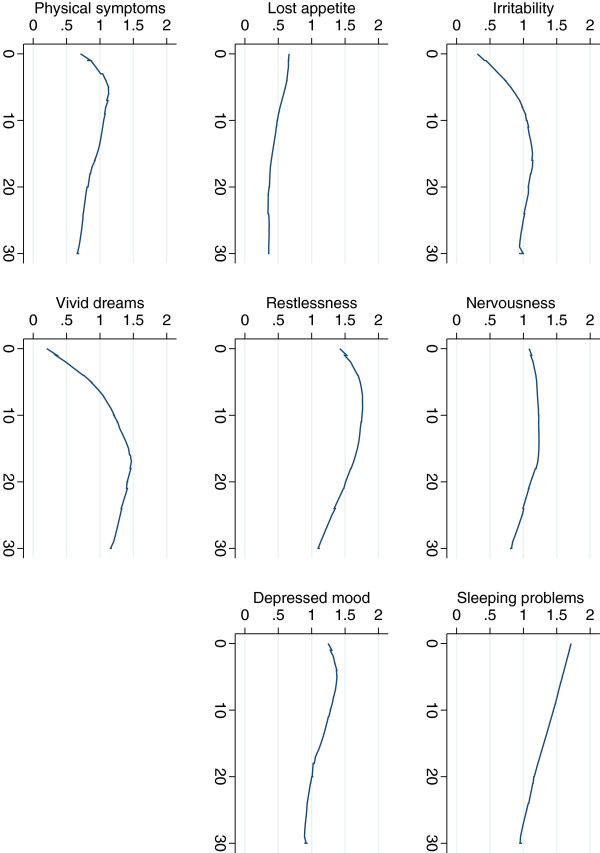
**Symptom severity for cannabis withdrawal symptoms as a function of time since last cannabis use: irritability, anxiousness, sleeping difficulties, reduced appetite, restlessness, feeling depressed, physical symptom and vivid dreams.** X-axis: days since last cannabis use. Y-axis: severity rating.

Fixed effects for individual symptoms are summarized in Table 4. For five of seven symptoms there was a significant linear decline with days elapsed since cannabis cessation: nervousness, insomnia, restlessness, depressed mood, and physical symptoms. For four of seven symptoms, there was a significant curvilinear relationship, indicating in all cases but one that symptoms first increased and then declined: irritability, nervousness, insomnia and restlessness. For insomnia, there was a significant curvilinear effect, but the effect was based on a linear decline and a positive quadratic effect, suggesting that with time the decline became weaker. However, neither the linear nor the quadratic coefficient was significant, and curvilinearity was not evident from inspection of the graph. The course of specific symptoms as assessed with LOWESS is illustrated in Figure 3.

**Table 4:** Fixed effects slopes for individual symptoms

		**Linear**			**Quadratic**			
**Symptom:**		**Beta**	**SE**	**F-test (1,145)**	**Beta**	**SE**	**F-test (2,144)**	**Additional substances**
Irritability/anger	Linear	0.001	0.001	0.03	**0.089**	**0.03**	*4.53	
	Squared				**-0.003**	**0.001**		
Nervousness	Linear	**-0.024**	**0.008**	**9.18	0.025	0.030	**6.08	
	Squared				-0.014	0.008		
Insomnia	Linear	**-0.036**	**0.008**	***18.76	-0.042	0.031	***9.34	Stimulants +
	Squared				0.000	0.000		
Loss of appetite	Linear	-0.006	0.006	1.01	-0.029	0.022	1.09	Opioids -
	Squared				0.001	0.001		
I am restless	Linear	**-0.024**	**0.008**	**9.66	0.009	0.029	**5.54	
	Squared				-0.001	0.001		
I feel depressed	Linear	*-0.016*	*0.007*	*5.37	-0.014	0.027	2.67	
	Squared				-0.000	0.001		
Physical symptoms	Linear	*-0.015*	*0.007*	*4.73	-0.034	0.027	2.61	
	Squared				0.001	0.001		
Vivid dreams	Linear	0.017	0.009	3.35	**0.114**	**0.033**	**6.30	
	Squared				**-0.003**	**0.001**		

All effects controlled for three additional substances (all time-variant variables): Days since last alcohol or benzodiazepine use; Days since last opioid use; Days since last CS use.

Coefficients in bold are significant at p < 0.01, coefficients in italics are significant at p < 0.05.

F-tests for linear model test that the linear effect of time since last use of cannabis is significant. F-tests for quadratic models test that the combined effect of the linear and the squared effect of days since last use of cannabis is significant. * p < 0.05. ** p < 0.01. *** p < 0.001.

Only loss of appetite was not associated with either a linear decline or a curvilinear function in time elapsed since last use of cannabis in this study.

Two of the symptoms that originally intended to measure cannabis withdrawal were associated with other substances in the analyses: insomnia was positively associated with time elapsed since last use of stimulants, and loss of appetite declined significantly with time elapsed since last use of opioids.

To compare the findings with regard to time of onset, we entered the day of peak of symptoms in Table 5 with the only published study in which the peak of individual symptoms was reported [11]. Although the results were not identically, both studies indicated that irritability/anger and vivid dreams had a later onset than other symptoms.

**Table 5:** Time course descriptions

	**Peak in days since last cannabis use**	**Peak from Budney study [**[11]**]**
Irritability/anger	14	^1^8,33
Nervousness	4	9
Insomnia	1	2
Loss of appetite	5	2
Restlessness	6	4
Depression	5	Not reported
Physical symptoms	5	^2^1,5
Vivid dreams	11	9

Notes:

^1^Averaged over 3 items.

^2^Averaged over 2 items.

#### ***The impact of medications***

The impact of antipsychotics and antidepressants was tested by running the regression analyses a second time, this time adding dummy variables representing antipsychotics and antidepressants to the equation, i.e. when any subject reported receiving a medication at any point this was added as a covariate to the equation. This analysis was repeated for each type of medication. In no instance did medications predict severity of any symptom or the full withdrawal scale or change the results.

#### ***Symptoms of withdrawal from other substances***

The remaining 19 symptoms (i.e., symptoms of opioid, alcohol and stimulant withdrawal) listed in the questionnaire were subjected to the same analyses as the full withdrawal scale. These symptoms were phrased to identify symptoms of withdrawal from cocaine, amphetamine, opioids, alcohol and tranquilizers. One symptom, vivid unpleasant dreams, showed evidence of a curvilinear association with time elapsed since last use of cannabis, and the Lowess graph is shown in Figure 3.

Another symptom, increased appetite (item 11) increased linearly since last use of cannabis. Finally, two items, fatigue (item 8) and yawning (item 14) showed evidence of a curvilinear relationship to time since last use of cannabis, with an increase during the first days, followed by a decline.

## Discussion

This is the first study to assess the DSM-5 cannabis withdrawal syndrome as described in the coming manual. The findings lend support to the new construct, and support its conceptualization in the DSM-5.

The study also supports that cannabis withdrawal syndrome is partly independent of other drug use. Cannabis withdrawal followed a clear time-course consistent with other research with a peak in overall severity 10 days after last use, followed by a gradual decline over the next 20 days e.g. [9]. This finding was evident, even though we had attempted to reduce demand characteristics by not informing patients that a focus of the study was on cannabis withdrawal.

Out of the seven symptoms listed in the DSM-5, five showed a linear decrease with time since last use of cannabis, and four showed evidence of a curvilinear course of symptoms with increasing symptoms in the early days, and declining symptoms in the later days. Evidence of a linear or curvilinear relationship between days since last cannabis use was found for only four of 19 symptoms of withdrawal from other substances. One of these symptoms, vivid dreams has been shown to be related to cannabis withdrawal in previous research [9,11,30]. It is likely that two of the other symptoms, fatigue and yawning, both may be influenced by patients experiencing insomnia or sleeping problems.

The time course of individual self-reported withdrawal symptoms largely mirrored what was observed in the study by Budney and colleagues [11]. The immediate symptoms that peaked within the first week after cessation in both studies included insomnia, loss of appetite, physical symptoms and restlessness. In the later phase of withdrawal came irritability/anger, and vivid, unpleasant dreams, with peaks later than a week after cessation.

One symptom differed between this study and Budney and colleagues’ study, namely nervousness, which had an immediate onset in this study, but peaked only after 9 days in Budney and colleagues’ study. This symptom was not associated with other drugs reported in our study, so at present we cannot explain this inconsistency. And on the other hand, visual inspection of Figure 1 in the study by Kouri and Pope suggests that in their study, anxiety peaked almost immediately [23].

The criterion of decreased appetite was not significantly associated with time since last cannabis use. It was however associated with time since last use of opiates, but was rated quite mildly in the patient sample as a whole. In contrast, patients in this study reported gradually increasing appetite over the whole 30 days since their last cannabis use.

Overall, this study gave very considerable support to the DSM-5 construct. The findings also give further support to the inclusion of the criterion of vivid unpleasant dreams, not currently included in the definition.

The consistent finding that the time course of symptoms varies may be caused by a wide range of underlying mechanisms. Some symptoms may occur as soon as cannabis use ceases and blood-levels of THC and other active components in cannabis begin to drop. Other symptoms may not occur until THC is nearly completely eliminated from the blood stream. For instance, in the study by Kouri and Pope, it appears that self-reported increased anxiety, increased physical tension and appetite loss begins immediately after cessation, but that irritability low mood and other physical symptoms accelerate somewhat more slowly as THC levels drop further [23]. Similarly, we found that nervousness increased immediately after cessation, and that irritability showed an initial increase followed by a decrease. For low mood, we found conflicting evidence, with low mood declining linearly from cessation in our study.

It is likely that medications affect the time course of withdrawal symptoms. However, this study found that neither antipsychotics nor antidepressants changed the results of severity of symptoms across time. Some of the mechanisms may also be psychological factors such as expectations to sensations after cessation of drug use. Explaining this variability may yield important information for the understanding of the underlying mechanisms in the cannabis withdrawal syndrome.

Another interesting option is to either maintain or taper patients on THC-based medications [14] or medications directed at particular symptoms such as sleep disturbances [16].

### Strengths and limitations

This study had some limitations that must be acknowledged. We were not able to ensure that participants completed the questionnaires at pre-fixed times since their last use of cannabis. Instead, we had to rely on self-reported time since their last cannabis use. Additionally, we did not assess severity of dependence, a factor that has been shown to be very important in predicting who will develop significant withdrawal, more so than frequency of use or amounts [9]. Further, we did not know the amount of each drug consumed, or the duration of time that the patients had used various substances. This could potentially affect the withdrawal symptoms associated with various substances, and this is a limitation that needs to be assessed in future studies of withdrawal in patients with poly-substance use disorders.

A further limitation is that the sample size is not very large. Interpretations concerning individual symptoms should be done in light of the existing literature, rather than based on significance testing, given that this study included multiple tests.

We were not able to collect baseline data from the patient sample while patients were still using cannabis or other drugs and alcohol. In spite of this, the time course described in both the graphical and statistical analyses of the data was remarkably similar to the time course in at least two previous studies where baseline data included current drug use - one for individual symptoms [11], and one for the total severity of symptoms [9].

Although we attempted to reduce the probability of demand characteristics in the symptom withdrawal questionnaire, patient ratings may be affected by the fact that it was administered at the same time that the participants were questioned about their drug cessation status. Also, it cannot be out ruled that the data collection was affected by the treatment providers at the treatment institutions. An additional limitation of the study is that we were not able to obtain a sample of all patients who were consecutively admitted to treatment. However, a comparison of our sample with the general patient population in residential treatment institutions in Denmark in the same period showed that the patient sample in this study was comparable in terms of age, gender composition, types of drug use and problem severity.

Urine control with our patients was quite sporadic, but given that the patients were in residential rehabilitation centers, there is a low risk that the patients would have access to drugs during their stay. We do, however, acknowledge the potential risk that the treatment providers that collected the data for this study could have influenced patient reports on drug use.

A further limitation is that we do not know to which extent findings concerning different symptoms may have been associated with other life stressors, including the particular stress associated with having arrived at a residential treatment institution and being removed from friends or family. It is possible that this might create a temporary increase in stress over the first few days of treatment, or that patients become increasingly aware of both physical and psychological symptoms as they move away from the hassles of the drug using lifestyle. Further research is needed to disentangle this relationship.

### Clinical implications

This study and other studies on cannabis withdrawal symptoms stress the need for developing interventions that target symptoms related to cannabis withdrawal. Limited evidence is available concerning pharmacological treatment [13,31]. Some clinicians recommend psychoeducation [32], but we know of no studies of the usefulness of this approach. Further research is needed to identify efficacious behavioral or pharmacological interventions.

## Conclusion

The findings yield strong support to the notion of a specific cannabis withdrawal syndrome. With the exception of loss of appetite, cannabis withdrawal symptoms in this study were associated with all types of withdrawal symptoms listed in the DSM-5. Findings in this study give further evidence for the inclusion of the criterion of vivid, unpleasant dreams, not listed in the current DSM-5 proposal, and challenge the significance of demand characteristics in determining the course of the symptoms of cannabis withdrawal.

## Competing interests

Both authors have no actual or potential conflict of interest including any financial, personal or other relationships with other people or organizations within three (3) years of beginning the work submitted that could inappropriately influence (bias) our work.

## Authors’ contributions

Both MH and BT designed and carried out the study. Both authors conducted literature searches and provided summaries of previous research studies. MH conducted the statistical analysis. Both authors wrote the first draft of the manuscript and both authors contributed to and have approved the final manuscript.

## Pre-publication history

The pre-publication history for this paper can be accessed here:

http://www.biomedcentral.com/1471-244X/13/258/prepub
